# Correlates of competitive anxiety in student athletes: the interactive roles of competitive stress, psychological resilience, and achievement motivation

**DOI:** 10.3389/fpsyg.2026.1832383

**Published:** 2026-05-22

**Authors:** Yin Chen, Yan Bu, Chen Sun, Dae Hee Kim

**Affiliations:** 1 College of Physical Education and Health Science, Yibin University, Yibin, Sichuan, China; 2 Department of Marine Sports, Pukyong National University, Busan, Republic of Korea

**Keywords:** achievement motivation, college student athletes, competitive anxiety, competitive stress, psychological resilience

## Abstract

**Background:**

Competitive anxiety (CA) is a common mental health concern among college student athletes. Although competitive stress (CS) is an important correlate of CA, the roles of psychological resilience (PR) and achievement motivation (AM) in this association remain unclear. Guided by Conservation of Resources theory, this study examined whether PR and AM mediated the relationship between CS and CA.

**Methods:**

A cross-sectional survey was conducted among 801 college student athletes from three universities in China (42.82% male, 57.18% female; most aged 18–22 years). Participants completed the Perceived Stress Scale, the Chinese revised Connor–Davidson Resilience Scale, the Achievement Motivation Scale, and the Sport Anxiety Scale-2. Regression-based mediation analysis and multi-group analysis were performed, controlling for gender and training experience.

**Results:**

CS was positively associated with CA (*r* = 0.750, *p* < 0.001) and negatively associated with PR (*r* = −0.611, *p* < 0.001) and AM (*r* = −0.640, *p* < 0.001). PR and AM independently mediated the relationship between CS and CA, and also formed a significant chain mediation effect. The negative association between CS and PR was stronger among male athletes (Δ*β* = −0.144, *p* = 0.048).

**Conclusion:**

CS was directly and indirectly associated with CA through PR and AM. These findings suggest that coaches should help athletes reduce CS, and incorporate PR training, goal setting, and AM support into regular psychological preparation to better manage CA.

## Introduction

In recent years, with the rapid development of competitive and mass sports, the mental health of athletes has increasingly attracted attention from both scholars and the public (Schinke et al., 2024; Tamminen et al., 2025). Among related topics, competitive anxiety (CA) has become a central issue in sport psychology because of its significant impact on athletes’ psychological states and performance (Domínguez-González et al., 2024; Yang et al., 2024). In the sports context, CA refers to the anxiety that manifests in athletes immediately preceding or while engaging in competitive events (Martens, 1977). Ideally, competitive anxiety should remain at a manageable level and be regulated effectively so that athletes can maintain emotional stability, attentional focus, self-confidence, and consistent performance under competitive pressure. Previous studies have shown that CA not only influences athletes’ psychological conditions but also negatively affects cognitive functioning, attentional focus, and athletic performance (Aliberti et al., 2024; Kaplánová, 2024; Tomé-Lourido et al., 2023). If CA is not identified and addressed in a timely manner, its negative effects may accumulate across training and competition, resulting not only in impaired performance but also in reduced self-confidence, maladaptive coping, and increased vulnerability to longer-term psychological difficulties. Therefore, clarifying the factors associated with CA is an urgent issue in collegiate sport settings.

In China, with the advancement of the sports industry and the improvement of athletic standards, college student athletes are exposed to multiple sources of stress, including the demands of competitions, expectations from coaches and teams, scrutiny from audiences and the public, as well as health- and injury-related challenges (Wang et al., 2024). Particularly in high-level competitive contexts, athletes often experience elevated psychological pressure and anxiety, which can undermine training effectiveness and performance while heightening susceptibility to psychological difficulties (Bürger et al., 2025; Liu et al., 2024). Moreover, focusing specifically on Chinese college student athletes is important because their competitive experiences are shaped by a performance-oriented sport environment and a distinctive combination of training, academic demands, and social expectations. Without a clearer understanding of the factors associated with CA in this group, opportunities for early identification, targeted support, and context-sensitive intervention may be missed.

When examining the mechanisms underlying CA in college student athletes, increasing attention has been given to the interaction between external stressors and individual psychological resources. Competitive stress (CS), which stems from performance outcomes, coaches’ evaluations, and peer comparisons, has been consistently identified as a key contributor to CA (Klingner et al., 2023; Yoon et al., 2022). At the same time, psychological resilience (PR), recognized as a vital personal resource for adapting to challenges and maintaining mental balance, has been shown to buffer the effects of CA (Simon et al., 2025; Yu et al., 2024). Achievement motivation (AM), serves as a crucial internal drive that encourages individuals to pursue excellence and overcome challenges, and it significantly influences emotional and behavioral responses in competitive settings (Gabrys and Wontorczyk, 2023; Kaplánová, 2024). Taken together, CS, PR, and AM may jointly constitute a key psychological mechanism influencing CA among college student athletes.

Although previous studies have examined the relationships among CS, PR, AM, and athletes’ psychological well-being, several research gaps remain. First, much of the prior literature has examined the separate or direct effects of these variables, whereas their integrated roles in explaining CA remain underexplored. Second, although PR and AM have each been linked to anxiety-related outcomes, few studies have examined whether they function as sequential mediators between CS and CA. Third, there is still a dearth of empirical data on Chinese collegiate athletes, especially when it comes to whether these pathways vary by gender. The current study uses the Conservation of Resources (COR) theory (Hobfoll, 1989) to fill these gaps by testing a chain mediation model in which CS predicts CA through PR and AM and using multi-group analysis (MGA) to look at gender variations in these associations.

## Literature review and hypotheses

### Conservation of resources theory

COR theory, proposed by Hobfoll (1989), posits that individuals strive to obtain, maintain, and protect valued resources, and that psychological distress is likely to arise when these resources are threatened or lost. In this framework, stress is understood primarily as a process of resource loss rather than simply as a direct reaction to external demands (Hobfoll et al., 2018). In competitive sport contexts, CS can be viewed as an external threat that may deplete athletes’ internal resources, thereby increasing their vulnerability to CA (Wolf and Utesch, 2024). Among these resources, PR helps athletes maintain stability and adapt under pressure, whereas AM supports persistence in goal-directed behavior (Alismail et al., 2025; Li and Pan, 2025). Accordingly, COR theory provides an appropriate framework for understanding how CS may be associated with CA through changes in PR and AM among college student athletes.

### Competitive stress and competitive anxiety

CS refers to the external pressure athletes perceive during competition due to performance goals, expectations from coaches and peers, external evaluations, and social comparisons (Mellalieu et al., 2009). In sport psychology research, CS has been widely regarded as a key antecedent of CA (Klingner et al., 2023; Yoon et al., 2022). For instance, Klingner et al. (2023) indicated that athletes exposed to intense CS tend to experience worry and unease, which in turn elevates anxiety. Moreover, Shi et al. (2024) found that intense CS significantly undermines athletes’ attentional focus and cognitive control, leading to cognitive and somatic anxiety before competitions. Taken together, existing studies consistently suggest that CS is an important antecedent of CA. However, further evidence is still needed to verify whether this relationship holds among Chinese college student athletes. Therefore, the following hypothesis is proposed:

*H1*: CS is positively associated with CA among college student athletes.

### The mediating role of psychological resilience

In sports activities, PR is commonly defined as athletes’ capacity to maintain psychological stability and respond positively when facing challenges such as competitive pressure, failure, or injury (Predoiu et al., 2025). Prior research has demonstrated that higher PR is closely associated with lower CA (González-Hernández et al., 2020; Yu et al., 2024; Zhang et al., 2024). For example, Raper et al. (2024) indicated that PR can helps athletes decrease anxiety and strengthens confidence and concentration, thereby alleviating anxiety-related performance impairment. Thus, PR, as a key internal resource, may play a protective role in the development of CA.

Research has also suggested that CS may weaken PR (Dwihandaka et al., 2024; Endo et al., 2023). Gong et al. (2023) reported that individuals under pressure are more likely to experience resource depletion, which reduces their resilience. Ma et al. (2025) further argued that prolonged external stress undermines emotional regulation and adaptability, making individuals more prone to negative reactions when coping with stress. However, although resilience has been associated with both stress and anxiety, its mediating role in this relationship has not been sufficiently examined among college student athletes. Therefore, the following hypothesis is proposed:

*H2*: PR mediates the relationship between CS and CA among college student athletes.

### The mediating role of achievement motivation

AM refers to the psychological process that stimulates, directs, and sustains behavior and performance (Roberts et al., 2007). According to sport psychology, AM is a significant psychological component that motivates athletes to engage in active training and competition (Krasmik et al., 2024). Previous studies have indicated that higher AM is often closely associated with lower anxiety (Ma and Hu, 2025; Wang, 2021). For instance, Ma and Hu (2025) found that individuals with AM tended to maintain goal orientation and active engagement, thereby reducing anxiety during competitions. Similarly, Dong et al. (2024) showed that AM enhances self-efficacy and persistence, enabling individuals to display greater psychological stability under high-pressure conditions and consequently alleviating anxiety responses.

On the other hand, studies have suggested that CS may undermine AM (Antonio, 2023; Morbée et al., 2025). Morbée et al. (2025) noted that individuals exposed to prolonged CS are more likely to experience frustration and helplessness, which consequently weakens their AM. Zhao et al. (2025) also found that when individuals view their objectives as hard to attain under high-pressure conditions, their level of AM significantly decreases. However, much of the prior literature has examined direct associations, while the mediating role of AM in this relationship remains underexplored. Therefore, the following hypothesis is proposed:

*H3*: AM mediates the relationship between CS and CA among college student athletes.

### The chain mediating role of psychological resilience and achievement motivation

PR has been shown to exert a significant positive effect on AM (Ding et al., 2025; Li and Pan, 2025). Individuals with PR often exhibit stronger motivation and are more inclined to demonstrate persistence and a self-improvement orientation in training and competition (Abdolrezapour et al., 2023; Hu et al., 2025). In contrast, insufficient resilience makes individuals more likely to lose confidence and goal orientation under stress, thereby weakening their motivation (Yıldırım et al., 2023). According to the COR theory (Hobfoll, 1989), external stress undermines key psychological resources, and changes in these resources further affect motivation, ultimately influencing emotional and behavioral responses. Taken together, PR and AM may jointly form a chain mediation pathway between CS and CA. Taken together, PR and AM may operate in a sequential manner, jointly linking CS to CA. However, few studies have examined this integrated chain mediation mechanism within a single model, particularly among Chinese college student athletes. Therefore, the following hypothesis is proposed:

*H4*: PR and AM jointly serve as a chain mediator in the relationship between CS and CA among college student athletes.

**Figure 1:**
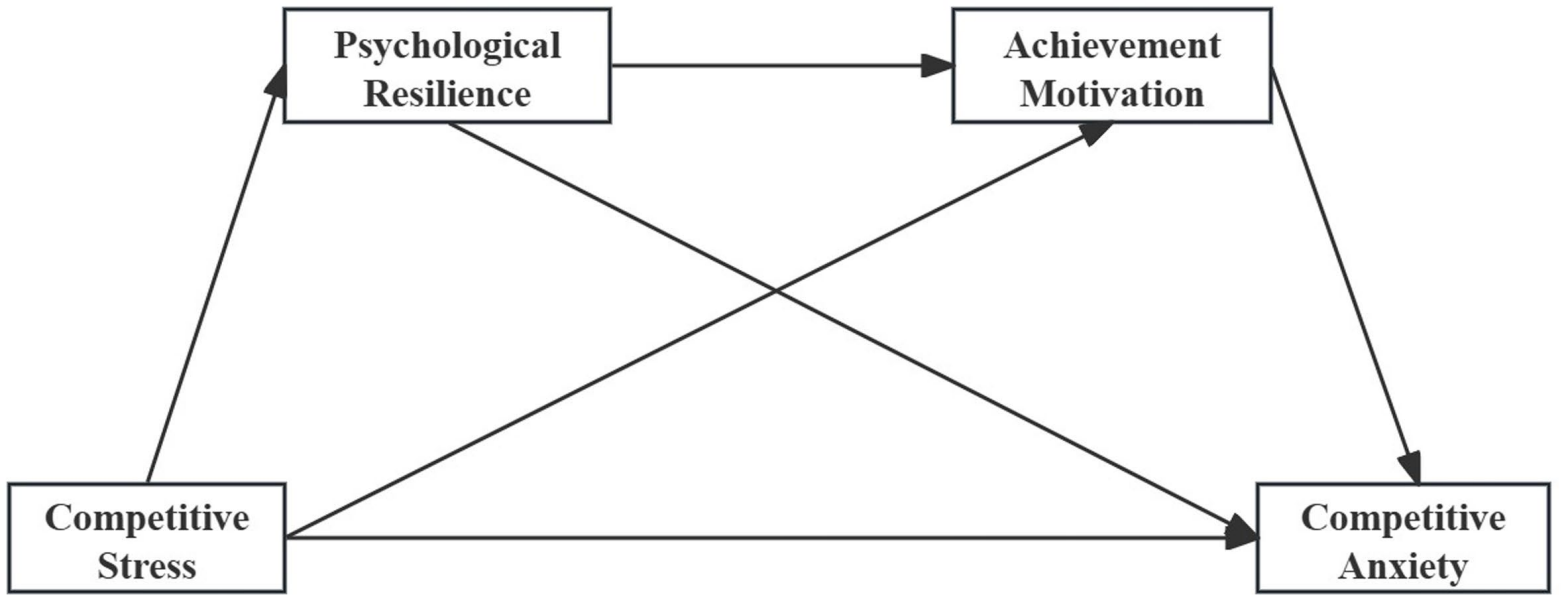
Hypothesized model.

The theoretical model of this study is shown in Figure 1.

## Methods

### Research design

This study employed a cross-sectional questionnaire survey design to examine college student athletes’ competitive stress, psychological resilience, achievement motivation, and competitive anxiety. The use of a questionnaire approach was justified because the focal variables reflect athletes’ subjective psychological experiences and motivational tendencies, which are appropriately quantified using standardized self-report scales to ensure measurement consistency and comparability, and to align with the operationalization requirements of Conservation of Resources theory. With respect to data analysis, given that the proposed model involves multiple psychological variables and a chain mediation mechanism, we employed regression-based mediation analysis with bootstrapping to test the indirect effects, and used multi-group analysis to examine whether the structural paths differed by gender.

Although the original questionnaire items were ordinal, the main analyses in this study were conducted on composite scores derived from validated multi-item scales. Methodological research has suggested that parametric statistical procedures are generally acceptable for summated Likert-type data when the scale scores are aggregated across items, the sample size is sufficiently large, and the score distributions are not severely non-normal (Harpe, 2015). In the present study, the sample size was large, and the composite scores showed acceptable skewness and kurtosis. Therefore, Pearson correlations and regression-based mediation analyses were retained. In addition, indirect effects were tested using bootstrap confidence intervals, which reduce reliance on normality assumptions.

### Participants and procedures

#### Participants and data collection

Three Chinese universities—Yibin University, Sichuan University of Science and Engineering, and Chengdu International Studies University—provided data for this study between February and March of 2026. A two-stage sampling procedure was used. First, three universities were selected as the study sites. Second, after obtaining the lists of enrolled college student athletes from each university, participants were randomly selected using a random number generator and invited to complete an online questionnaire. To ensure privacy, all questionnaires were collected anonymously through the Wenjuanxing platform, which did not record any identifiable information. Participants were made aware that participation in the survey was completely voluntary and that they could opt out at any point without facing any repercussions. Only the study team had access to the securely kept data, which was utilized exclusively for research. Every participant gave their informed consent prior to the survey. Additional consent was acquired from the legal guardians of participants under the age of eighteen. The Yibin University Ethics Committee gave its ethical approval.

To determine an appropriate sample size, the study followed Kline (2018) and set a planned sample size of 780 after allowing for a potential 20% attrition rate. Furthermore, an *a priori* power analysis using G*Power 3.1 (Faul et al., 2009) revealed that a minimum sample size of 160 was needed with *f*^2^ = 0.15, *α* = 0.05, 1 — *β* = 0.95, and eight predictor variables. Of the 850 questionnaires distributed, 823 were returned, yielding a response rate of 96.8%. After excluding 22 invalid questionnaires due to missing data or careless responses, 801 valid responses were retained for analysis.

### Inclusion and exclusion criteria

Participants were eligible for inclusion if they: (a) were full-time college student athletes currently enrolled in one of the three participating universities, (b) were able to complete the online questionnaire independently, and (c) provided informed consent; for participants under 18 years of age, guardian consent was also required. Participants were excluded if they: (a) did not meet the above inclusion criteria, (b) submitted questionnaires with substantial missing data, or (c) provided careless or invalid responses. After data screening, 22 questionnaires were excluded on these grounds.

### Demographic characteristics and preliminary analyses

The demographic characteristics of the participants are summarized in Table 1. Gender distribution was fairly balanced, with 343 males (42.82%) and 458 females (57.18%). 90.64% of participants were between the ages of 18 and 22, with 18–20 years accounting for 52.43% and 20–22 years for 38.21%, which reflects the typical age range of college students. Regarding grade level, freshmen, sophomores, and juniors were the main groups, accounting for 26.34, 33.71, and 31.34%, respectively, while seniors accounted for 8.61%. With respect to training experience, 55.31% had trained for 0–1 year, 41.57% for 2–3 years, and only 3.12% for more than 4 years. Concerning sport type, ball sports (22.53%), water sports (22.16%), gymnastics (16.17%), and ice and snow sports (16.04%) were the most common, followed by track and field (13.42%), strength sports (6.87%), and extreme sports (2.81%).

**Table 1:** Demographic characteristics of the sample and differences in CA.

Variables	Category	Frequency	Percentage	CA (M ± SD)	T/F	*p*
Gender	Male	343	42.82%	2.569 ± 0.472	5.196*	0.023
	Female	458	57.18%	2.610 ± 0.409		
Age	<18	40	4.99%	2.533 ± 0.485	2.323	0.074
	18–20	420	52.43%	2.610 ± 0.415		
	20–22	306	38.21%	2.596 ± 0.428		
	>22	35	4.37%	2.419 ± 0.648		
Grade	Freshman	211	26.34%	2.628 ± 0.424	1.799	0.146
	Sophomore	270	33.71%	2.543 ± 0.436		
	Junior	251	31.34%	2.611 ± 0.437		
	Senior	69	8.61%	2.611 ± 0.475		
Training years	0–1 Year	443	55.31%	2.670 ± 0.345	56.749***	<0.001
	2–3 Years	333	41.57%	2.550 ± 0.443		
	≥4 Years	25	3.12%	1.797 ± 0.823		
Sport type	Track and Field	108	13.42%	2.499 ± 0.519	1.480	0.182
	Ball Sports	181	22.53%	2.591 ± 0.462		
	Water Sports	178	22.16%	2.615 ± 0.406		
	Ice and Snow Sports	129	16.04%	2.656 ± 0.367		
	Gymnastics	130	16.17%	2.601 ± 0.430		
	Strength Sports	52	6.87%	2.547 ± 0.455		
	Extreme Sports	23	2.81%	2.562 ± 0.383		

***p < 0.001, *p < 0.05.

Independent-samples t-tests and one-way analysis of variance (ANOVAs) were performed on the main study variables in order to better investigate the impact of demographic characteristics on CA and lessen the interference of individual differences in the results. The results showed that age, grade level, and sport type had no significant effect on CA. The effect sizes for age, grade level, and sport type were small (*η*^2^ = 0.009, 0.007, and 0.011, respectively), whereas training experience showed a moderate effect size (*η*^2^ = 0.125). However, gender and training experience exerted significant effects, which were controlled for in subsequent analyses.

### Measures

CS was assessed using the Perceived Stress Scale originally developed by Cohen et al. (1983) and revised by Liu et al. (2023). The scale contains 10 items, including 4 reverse-scored items. Higher scores indicate greater perceived CS among college student athletes. In the present study, Cronbach’s *α* was 0.904, *χ*^2^/df = 3.153, CFI = 0.979, GFI = 0.973, TLI = 0.973, and RMSEA = 0.052. PR was measured with the Chinese revised version of the Connor–Davidson Resilience Scale originally developed by Connor and Davidson (2003) and revised by Su and He (2023). This unidimensional scale consists of 10 items, with higher scores reflecting stronger resilience. In the present study, Cronbach’s α was 0.911, *χ*^2^/df = 1.465, CFI = 0.996, GFI = 0.987, TLI = 0.994, and RMSEA = 0.024. AM was assessed using the AM Scale originally developed by Ye and Hagtvet (1992) and revised by Li et al. (2023). The scale includes two dimensions, pursuit of success and avoidance of failure, with a total of 30 items. Higher scores indicate stronger AM. In the present study, Cronbach’s α was 0.964, *χ*^2^/df = 2.893, CFI = 0.946, GFI = 0.916, TLI = 0.942, and RMSEA = 0.049. CA was measured with the Sport Anxiety Scale-2, originally developed by Smith et al. (2006)and revised by Zhang et al. (2023). The scale consists of three dimensions, somatic anxiety, worry, and concentration disruption, with a total of 15 items. Higher scores reflect higher levels of CA. In the present study, Cronbach’s α was 0.933, *χ*^2^/df = 3.049, CFI = 0.969, GFI = 0.957, TLI = 0.964, and RMSEA = 0.051.

### Data analysis

AMOS 24.0 and SPSS 26.0 were used for data analysis. Descriptive statistics were first calculated for every study variable. Skewness and kurtosis were used to evaluate normalcy; adequate normality was indicated by absolute skewness values below 2 and absolute kurtosis values below 7 (Kline, 2005). Bivariate relationships between the primary variables were next investigated using Pearson correlation analysis; absolute r values of around 0.10, 0.30, and 0.50 were considered as moderate, medium, and large, respectively (Cohen, 1988). The measurement model’s structural validity was then assessed using a CFA in AMOS, and common method bias (CMB) was evaluated by adding a CLF. Several indices were used to assess the model’s fit: *χ*^2^/df values below 3.00, GFI, CFI, and TLI values of 0.90 or above, and RMSEA values below 0.08 were deemed to indicate an adequate to good fit (Fietzer et al., 2025). Tolerance and VIF values were used to further evaluate multicollinearity. Lastly, the mediating functions of PR and AM in the link between CS and CA were tested using the bootstrap approach.

## Results

### Descriptive statistics

All of the primary variables’ skewness and kurtosis values fell within permissible bounds (Table 2), suggesting that the data were roughly regularly distributed.

**Table 2:** Descriptive statistics and normality statistics of the main variables.

Variables	*N*	M ± SD	MIN	MAX	SK	Kur
CS	801	2.922 ± 0.526	1	5	−0.151	3.323
PR	801	3.325 ± 0.626	1	5	−0.628	4.207
AM	801	2.542 ± 0.470	1	4	−0.270	4.823
CA	801	2.593 ± 0.437	1	4	−1.506	4.802

*N*, sample size; *M*, mean; SD, standard deviation; MIN, minimum; MAX, maximum; SK, skewness; Kur, kurtosis.

### Common method bias test

CLF was incorporated into the measurement model and connected to every observed variable in order to investigate common technique bias. The baseline model without the CLF and the model with the CLF were then contrasted (Podsakoff et al., 2003). CMB was not a major concern in this investigation, as indicated by the chi-square difference test’s lack of significance (*p* > 0.05). Furthermore, with *χ*^2^/df = 1.631, CFI = 0.958, GFI = 0.893, TLI = 0.956, and RMSEA = 0.028, the CFA results demonstrated a satisfactory fit of the measurement model.

### Correlation analysis

The correlations among CS, PR, AM, and CA were examined (Table 3). The results showed that CS was significantly negatively correlated with both PR and AM, and significantly positively correlated with CA. PR and AM were significantly positively correlated with each other, and both were significantly negatively correlated with CA. According to conventional guidelines for interpreting Pearson correlation coefficients, the observed associations were all large in magnitude.

**Table 3:** Correlation analysis of each independent variable.

Variables	CS	PR	AM	CA
CS	1			
PR	−0.611***	1		
AM	−0.640***	0.743***	1	
CA	0.750***	−0.731***	−0.749***	1

***p < 0.001.

### Collinearity test

To ensure the stability and explanatory validity of the model, collinearity diagnostics were conducted among the independent variables (Table 4). The results showed that the tolerance values of CS, PR, and AM were all greater than 0.1, and the VIF values were all below the critical threshold of 3.3 (Hair et al., 2019). Thus, multicollinearity was not an issue in this investigation.

**Table 4:** Collinearity diagnostics.

Variables	Tolerance	VIF
CS	0.549	1.821
PR	0.417	2.400
AM	0.393	2.547

### Construct reliability and validity

The measuring model’s validity and reliability were evaluated in accordance with Hair et al. (2021) recommendations (Table 5). The results showed that Cronbach’s *α* and CR values were well above the recommended threshold of 0.70, indicating good internal consistency. In addition, the AVE values surpassed the minimum standard of 0.50, suggesting that the model demonstrated good convergent validity.

**Table 5:** Construct reliability and validity.

Variables	Cronbach’s *α*	CR	AVE
CS	0.904	0.921	0.537
PR	0.911	0.926	0.557
AM	0.964	0.967	0.501
CA	0.933	0.941	0.517

CR, composite reliability; AVE, average variance extracted.

### Mediation analysis

**Figure 2:**
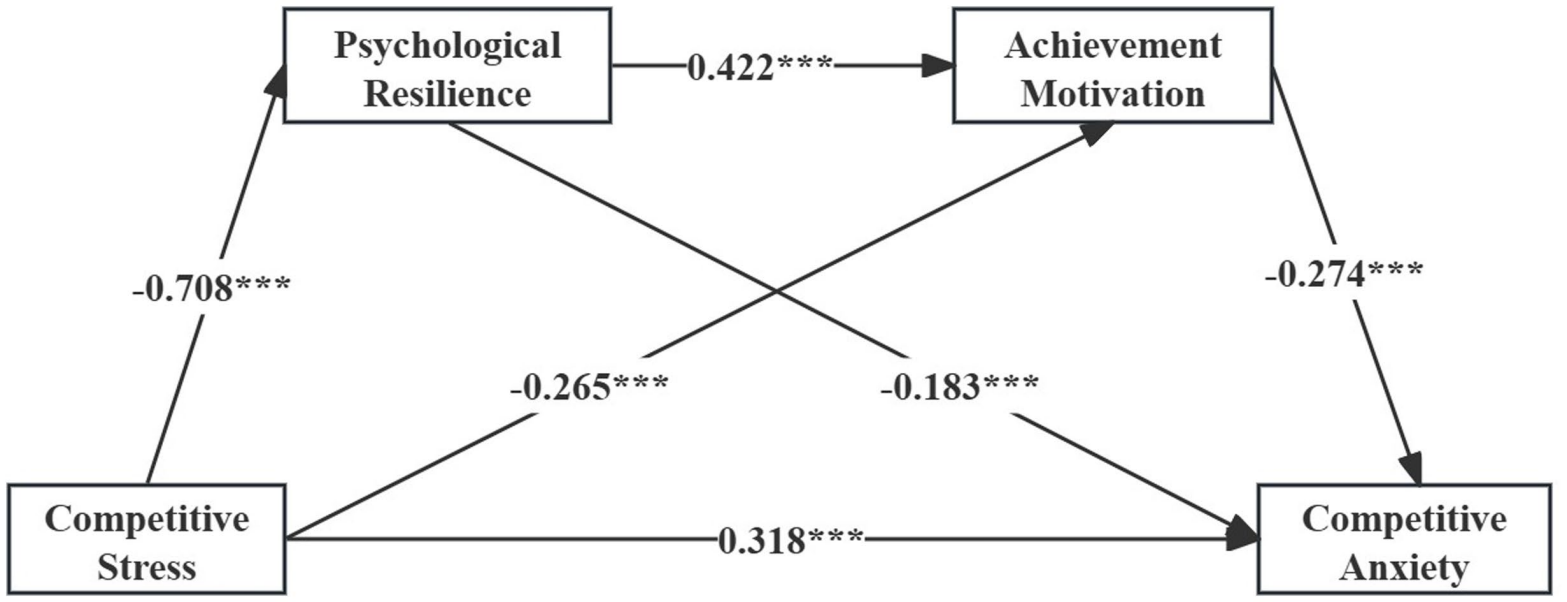
Path coefficient diagram.

After controlling for gender and years of training, CS negatively predicted PR and AM, and positively predicted CA (Table 6; Figure 2). PR positively predicted AM and negatively predicted CA, while AM also negatively predicted CA. Among the control variables, years of training was negatively associated with CA.

**Table 6:** Regression analysis.

Outcome variable	Predictor variable	*β*	SE	*T*	CI (95%)		*R* ^2^	*F*
					LLCL	ULCI		
PR	CS	−0.708***	0.034	−20.664	−0.775	−0.641	0.378	161.549
	Gender	−0.025	0.035	−0.705	−0.094	0.045		
	Training years	0.072*	0.032	2.240	0.009	0.135		
AM	CS	−0.265***	0.025	−10.455	−0.315	−0.215	0.607	307.929
	PR	0.422***	0.021	19.945	0.380	0.463		
	Gender	0.008	0.021	0.357	−0.034	0.049		
	Training years	0.002	0.019	0.113	−0.036	0.040		
CA	CS	0.318***	0.021	15.005	0.277	0.360	0.721	410.116
	PR	−0.183***	0.020	−9.016	−0.223	−0.143		
	AM	−0.274***	0.028	−9.844	−0.328	−0.219		
	Gender	−0.003	0.017	−0.184	−0.036	0.030		
	Training years	−0.059***	0.015	−3.902	−0.089	−0.029		

***p < 0.001, *p < 0.05.

The mediation analysis’s detailed findings are shown in Table 7. With the direct effect making up 52.8% of the total effect, CS had a large overall impact on CA. Crucially, none of the three indirect effects had zero in their 95% confidence intervals, making them all statistically significant. In particular, 21.5% of the overall benefit came from the indirect effect through PR, 12.1% from the indirect effect through AM, and 13.6% from the sequential indirect effect through both PR and AM.

**Table 7:** Mediation effect analysis.

Effect	Path	*β*	SE	CI (95%)		Percentage
				LLCI	ULCI	
Total effect	CS → CA	0.602	0.020	0.563	0.641	100%
Direct effect	CS → CA	0.318	0.021	0.277	0.360	52.8%
Indirect effect	CS → PR → CA	0.130	0.017	0.094	0.163	21.5%
	CS → AM → CA	0.073	0.015	0.047	0.107	12.1%
	CS → PR → AM → CA	0.082	0.015	0.056	0.114	13.6%

### Gender difference analysis

In order to investigate any measurement invariance problems, measurement invariance of composite models (MICOM) was investigated prior to performing MGA (Henseler et al., 2016). Measurement invariance was established across gender groups, according to the results (Table 8).

**Table 8:** MICOM compositional invariance: males vs. females.

Construct	Correlations (H 0 = 1)	5% Quantile	*p*	Compositional invariance
CS	1.0	1.0	0.393	Yes
PR	1.0	1.0	0.447	Yes
AM	1.0	1.0	0.597	Yes
CA	1.0	1.0	0.093	Yes

An MGA was carried out to investigate gender disparities. The male group experienced a substantially greater impact of CS on PR than the female group (Δ*β* = −0.144, *p* = 0.048) (Table 9). This finding suggests that, under competitive pressure, male student athletes may be more vulnerable to reductions in PR than female student athletes. From a practical perspective, this implies that stress-management and resilience-building interventions may need to place greater emphasis on male athletes, particularly in highly demanding competitive environments. At the same time, because no other structural paths showed significant gender differences, the overall model appears to be broadly similar across gender groups.

**Table 9:** MGA: males vs. females.

Path	Difference (male – female)	*p*	Significance
AM → CA	−0.071	0.387	No
CS → AM	−0.035	0.667	No
CS → CA	0.007	0.892	No
CS → PR	−0.144	0.048	Yes
PR → AM	−0.041	0.632	No
PR → CA	0.059	0.395	No

## Discussion

This study examined the relationships among CS, PR, AM, and CA in Chinese college student athletes. The findings supported all four hypotheses and further revealed a significant gender difference in the path from CS to PR.

This study found that CS was significantly positively associated with CA among Chinese college student athletes, supporting H1. This finding is consistent with Klingner et al. (2023) showing that stress in competitive settings increases worry, tension, and emotional instability. When athletes perceive strong external pressure, they may become more concerned about failure and evaluation, which increases self-doubt and anxiety (Gutiérrez-Capote et al., 2025; Yoon et al., 2022). In the Chinese collegiate sports context, athletes are often exposed to multiple sources of evaluation, including coaches, peers, and the broader performance-oriented environment (Xi et al., 2024). These conditions may further intensify the link between stress and anxiety. Therefore, intervention practices should focus on helping athletes better identify and regulate stress, for example through resilience training, cognitive reappraisal strategies, or social support systems, in order to buffer the negative impact of CS on CA.

PR mediated the relationship between CS and CA, supporting H2. This result is consistent with Yang et al. (2025), who demonstrated that stress can exacerbate anxiety by undermining resilience. High-intensity competitive environments may erode athletes’ sense of self-efficacy and control, thereby weakening resilience (Ma et al., 2025). A systematic review by Gupta and McCarthy (2022) synthesized 92 studies in sport and exercise psychology and emphasized that resilience should be understood as a sport-specific protective and adaptive process in response to adversity and evaluative pressure. Moreover, insufficient resilience makes individuals more prone to worry and self-doubt under stress, which increases experiences of CA (Dalmış et al., 2025). Therefore, universities and coaches should integrate resilience training and psychological support into daily practice to enhance athletes’ coping and emotion regulation abilities, thereby reducing the occurrence of CA.

AM mediated the relationship between CS and CA, supporting H3. These findings indicate that stress weakens individuals’ motivation, leading them to lose positive goal orientation and self-drive within training and competitive contexts (Huang et al., 2025). People often see competitive situations as threats rather than challenges when AM declines, which increases anxiety levels by causing worry, self-doubt, and tension (Han et al., 2022). A study of elite athletes in Iran found that sport orientation could significantly predict CA, and that higher competitiveness and goal orientation were associated with lower CA (Jamshidi et al., 2011). In addition, a recent systematic review and meta-analysis on motivational climate in youth sport suggested that motivational processes show their strongest effects on internal psychological outcomes, particularly motivation, cognition, and emotional well-being (Gu and Cheng, 2026). Therefore, fostering and maintaining athletes’ AM, and helping them develop positive goal pursuit and a success-oriented mindset, is of great importance for buffering the adverse impact of CS on anxiety.

PR and AM jointly served as a chain mediator between CS and CA, supporting H4. A possible explanation is that resilience helps athletes preserve adaptive functioning under stress, including emotional stability, perceived competence, and persistence in goal pursuit. When CS erodes resilience, athletes may experience greater difficulty regulating negative emotions and recovering from setbacks, while also becoming less confident in their ability to achieve desired outcomes (Kökçam et al., 2022). Such changes may weaken AM, because sustained motivation in competitive settings depends not only on goals themselves but also on individuals’ belief that they can continue striving effectively despite obstacles (Li and Pan, 2025). Once this motivational drive is reduced, competition tend to be appraised as threatening rather than challenging, which increases CA (Dong et al., 2024). Therefore, the present findings suggest that resilience may serve as an important psychological foundation for the maintenance of AM under stress, and that the depletion of these two resources may sequentially contribute to anxiety.

The results of the MGA demonstrated that the negative effect of CS on PR was significantly stronger in the male group than in the female group. This finding is consistent with Sánchez-Teruel et al. (2021), who observed that women often display slightly higher resilience than men in stressful contexts. Within the framework of COR theory, CS represents a threat to valued personal resources, and its impact may be stronger when individuals perceive greater pressure to protect performance-related self-worth (Hobfoll, 1989). In the context of Chinese competitive sports, male athletes may be more likely to experience competition outcomes as closely tied to expectations of success, advancement, and recognition (Peng et al., 2025). Under such conditions, CS may be more readily appraised as a threat to valued resources, thereby resulting in greater depletion of PR. By contrast, although female athletes also perform in demanding environments, relatively more diverse evaluation criteria may somewhat reduce the extent to which competitive pressure is concentrated on performance outcomes alone (Zhao, 2024). This may help explain why the negative association between CS and PR was weaker in the female group. Therefore, the present finding not only highlights a gender difference in the stress–resilience link, but also suggests that the effect of CS on psychological resources may depend on how competitive demands are socially and psychologically framed.

### Implications

#### Theoretical implications

By constructing a chain mediation model linking CS, PR, AM, and CA, this study extends the application of COR theory. Prior studies have primarily focused on the direct associations between CS and anxiety, while paying less attention to how resource depletion relates to motivational and emotional processes. By incorporating PR and AM as representative internal resources, this study reveals a structural pathway through which external stress is associated with athletes’ emotional outcomes via resource transmission, thereby broadening the explanatory power of COR theory in the context of competitive sports. Furthermore, the findings indicate that PR and AM functioned as distinct mediators between CS and CA, enriching the resource dimensions of COR theory. PR reflects individuals’ adaptive capacity to cope with resource loss, while AM represents the investment and drive of resources under goal orientation. Differentiating and linking these two constructs through a chain mediation model helps clarify how resources are related at both psychological and motivational levels, ultimately manifesting as emotional responses. Finally, this study provides empirical support for the loss spiral proposed by COR theory by validating the sequential association of resource depletion. The findings suggest that CS is associated not only with lower levels of psychological and motivational resources but also with higher levels of CA. Within the context of Chinese college student athletes, these results further highlight the potential role of sociocultural factors in the resource–motivation–emotion pathway, offering a new perspective on the cross-cultural application of COR theory in studies of CS and adaptation.

#### Practical implications

The findings of this study provide actionable implications for psychological support and intervention in collegiate competitive sports settings. First, for coaches and psychological counselors, CS, PR, and AM should be incorporated into routine athlete management rather than addressed only after anxiety problems emerge. For example, coaches may conduct brief pre-competition stress check-ins and integrate 10–15-min weekly psychological skills sessions into training schedules, focusing on relaxation techniques, positive self-talk, imagery rehearsal, and goal-setting exercises. Psychological counselors can provide targeted support for athletes who report high stress or anxiety, such as cognitive reappraisal training, exposure-based coping practice, and individualized resilience-building plans. These strategies may help athletes maintain emotional stability, confidence, and task focus under pressure. Second, for university administrators and policy makers, the present findings suggest the need to establish a more structured psychological support system for student athletes. Universities could introduce regular screening for CS and anxiety at key time points, such as the beginning of the season, before major competitions, and after important losses or injuries. In addition, institutions may offer semester-based workshops on resilience enhancement, motivation regulation, and stress management, while also developing referral pathways between coaches, counselors, and sports departments to ensure timely support for at-risk athletes. For small and medium-sized universities with limited resources, group counseling, online psychoeducational modules, and shared mental health services across departments may represent cost-effective ways to expand support coverage. Finally, for student athletes themselves, the findings highlight the importance of actively managing psychological resources in daily training and competition preparation. Athletes can strengthen resilience and maintain AM by setting short-term process goals, monitoring their emotional states through self-reflection logs, and building stable routines that combine training, recovery, and social support. Before competitions, they may use practical strategies such as breathing regulation, positive self-dialogue, and pre-performance routines to reduce stress reactions and maintain concentration. Over the longer term, participation in teamwork activities, psychological skills training, and help-seeking when stress becomes overwhelming may contribute to both improved performance and better psychological well-being.

### Limitations and future research directions

This study has several limitations. First, the cross-sectional design limits the strength of causal interpretation. Although the present analyses supported the hypothesized associations, gender differences, and chained mediation pattern among CS, PR, AM, and CA, data collected at a single time point cannot fully establish temporal ordering, directional effects, or causal mediation. Future research could adopt longitudinal designs with multiple waves of data collection to examine whether CS predicts subsequent changes in resilience, motivation, and anxiety over time. More specifically, two-wave or three-wave panel studies conducted across training periods or competition cycles may help clarify the temporal stability and direction of these relationships. Second, the data in this study were primarily based on self-reported questionnaires from college student athletes. Although reliability and validity tests supported acceptable measurement quality, the potential influence of social desirability, self-presentation bias, and CMB cannot be completely ruled out. Future studies may combine multiple data sources and methods, such as coach or peer ratings, behavioral observations, physiological indicators, and repeated assessments conducted before and after competitions. Such designs would help improve ecological validity and provide a more comprehensive understanding of athletes’ psychological responses in real competitive settings. Finally, the sample was limited to Chinese college student athletes, which may restrict the generalizability of the findings across age groups, competitive levels, and cultural contexts. Moreover, although participants were randomly selected from the lists of eligible student athletes, stratified random sampling was not used. Therefore, some subgroups, such as athletes with longer training experience or those from less common sport types, may have been underrepresented. Future studies should extend the present model to more diverse samples and adopt stratified random sampling based on gender, grade level, training experience, and sport type to improve sample representativeness and generalizability.

## Conclusion

This study aimed to explore the mechanisms underlying CA among college student athletes, with a particular focus on how CS relates to anxiety through PR and AM. The results demonstrated that CS was significantly positively associated with CA, and both PR and AM served as significant mediators. Specifically, resilience reflects individuals’ ability to maintain psychological balance and adapt in high-pressure contexts, whereas AM represents their drive to remain engaged and persistent in goal pursuit. These two factors function not only as independent mediators but also as sequential links in a chain, jointly revealing the multilayered connections between resources, motivation, and emotions. By extending the application of COR theory, this study highlights the relational mechanisms among CS, internal resources, and emotional experiences, offering new insights into the psychological adaptation of college student athletes in high-intensity sport settings. The findings also carry practical implications, suggesting that enhancing resilience, maintaining AM, and developing emotion regulation strategies can help athletes better manage CA.

## Data Availability

The raw data supporting the conclusions of this article will be made available by the authors, without undue reservation.
